# Short-Term Effects of Land Leveling on Irrigation-Related Some Soil Properties in a Clay Loam Soil

**DOI:** 10.1155/2013/187490

**Published:** 2013-06-12

**Authors:** Tekin Öztekin

**Affiliations:** Department of Biosystem Engineering, Agricultural Faculty, Gaziosmanpasa University, Tasliciftlik Campus, 60250 Tokat, Turkey

## Abstract

There are few studies conducted on the short-term effects of land leveling on soil water holding capacity. The objectives of this study were to analyze the short-term effects of land leveling on the magnitudes, variances, spatial variability, and distributions of surface (0–20 cm) and subsurface (20–40 cm) soil properties of bulk density, field capacity, permanent wilting point, water holding capacity and particle size fractions. The study was conducted in a 1.2 ha field with clay loam soil located on the low terraces of Yesilirmak River, Tokat, Turkey. According to the paired *t*-test results, water holding capacity, and bulk density significantly increased, while permanent wilting point (*P* ≤ 0.001) and field capacity (*P* ≤ 0.05) significantly decreased for surface soil due to land leveling. The reasons for the increases in WHC values in both cut and fill areas (29%, and 12%, resp.) of surface soil are look like the much more decreases in PWP values than those of FC values and the increases in BD values. The moderate positive linear relationship between the surface soil clay contents and cut depths through cut areas (*r* = 0.64) was also determined in this study.

## 1. Introduction

In Turkey, land consolidation projects have gained a speedy acceleration during the last decade. This trend will likely continue into the near future when we consider the government common agricultural policy. One of the main purposes of land consolidation projects is to provide the land a uniform grade compatible for both drainage and surface irrigation methods. In order to create a slight but uniform slope across a field with land leveling, most commonly, topsoil from spots of relatively high elevations are simply scrapped away (i.e., cut) and deposited (i.e., filled) in areas of relatively low elevations [1]. In other words, land leveling is the operation of shaping the surface of land to predetermined grades so that each surface slopes to a drain or is configured for efficient application of irrigation water. 

Land leveling alters the depth to soil horizons relative to the original soil surface and, thus, may cause unfavorable surface conditions after land leveling, depending on the nature of the surface horizons [2]. Deep tillage with the application of N- and P-rich fertilizers may be required following land leveling to help alleviate the poor soil physical conditions. In addition, land leveling is to be maintained routinely because tillage or inappropriate erosive discharges disturb land leveling and the uniformity of water distribution. 

The major problem with land leveling is the effect of removing the topsoil and its subsequent influence on plant growth. Reduced growth may occur on the fill areas, although the exposure of subsoil in the cuts is usually a more serious problem [3]. In some less common instances, all of the topsoils in the area to be graded are scrapped off and piled, and topographic variations at the top of the subsoil are smoothed out, and the topsoils spread back on the manipulated area [1, 4]. In this case the cost can be justified.

Land leveling improves uniformity of crop growth and yield. It conserves soil and water by creating slight, but uniform, slope gradients to improve drainage, to drive irrigation water across the field with a suitable flow velocity without erosion, to facilitate more even distributions of irrigation water, to improve the effectiveness of surface irrigation, and so forth. Besides some of the mentioned benefits, land leveling also has some disadvantages. First of all, land leveling is a severe soil disturbance that disrupts or alters the entire equilibrium among near surface soil properties.

 The spatial variability, distributions, changes, and relationships in soil physical, chemical, and biological properties as a result of land leveling were reported mostly by Brye et al. in a few studies [1, 2, 4–9]. Bulk density, sand, and clay contents significantly increased, while silt content significantly decreased, and the variances of soil physical properties were unaffected due to land leveling in a Stuttgart silt loam soil [1]. However, in another study by Brye et al. [8] at the same soil, increases in bulk density and clay percentage and decreases in sand and silt percentages were found. In addition, soil pH and EC values and the variance of soil pH in the top 10 cm increased due to land leveling [9]. In another study by Brye et al. [7], there was a significant increase in the EC values in the top 10 cm, while, significant decrease in the soil pH values due to land leveling was reported. Furthermore, Ramos et al. [10] reported that the changes in the particle size distribution of the fine fraction due to land terracing affected hydraulic conductivity, water retention capacity, and aggregate stability, as well as the relationships between all these variables. 

 To our knowledge, few studies have reported the effects of land leveling on the soil physical properties of field capacity, permanent wilting point, and water holding capacity in clay loam soils. Evaluations about the changes and variability in soil properties in this study will improve management capabilities to ensure maximum production from the graded fields at clay loam soils. Furthermore, we are going to try to relate the changes in soil properties to the process of grading through analyses of cut and fill areas separately. This may help engineers decide whether the designed leveling projects with determined depths of cut and fill are also appropriate in the sense of soil physical properties, and readers generalize the results to other fields and types of leveling. 

 The objective of this study was to analyze the short-term effects of land leveling on the magnitudes, variances, spatial variability, and distributions of surface (0–20 cm) and subsurface (20–40 cm) soil properties of bulk density (BD), field capacity (FC), permanent wilting point (PWP), water holding capacity (WHC), particle size fractions, pH, and soil electrical conductivity (EC) in a clay loam soil.

## 2. Materials and Methods

### 2.1. Site Description and Experimental Design

 A 1.2 ha (120 × 100 m) field, previously cropped to wheat, on Channel clay loam soil [11] at the Agricultural Research and Application Center of Gaziosmanpaşa University in Tokat, Turkey (40°20′N lat., 36°28′E long., elevation = 600 m from mean sea level) was chosen as the study site in Fall 2010. The study area is located at a junction between colluvial and alluvial materials. The channel clay loam has a deep soil profile, low permeability, and poor drainage. This soil is typically located on the low terraces of Yeşilırmak River and classified as an ustorthent and ustifluvent according to the soil taxonomy [12] for surface and subsurface soils, respectively. Before land leveling, the research field was slightly nonuniform sloped (<3%) from north to south, and almost leveled (<0.3%) in the west-east direction. In general, the research area has been used for the research experiments of Agricultural Faculty of Gaziosmanpaşa University to grow forage and cereal plants irrigated by surface irrigation methods. In order to facilitate statistical evaluation of the effects of land leveling on the magnitude, variability, and spatial distribution associated with soil properties, a 120 m wide by 100 m long study area covering a 30-point grid was established with sampling points spaced evenly 20 m apart just before the land leveling. The grid-sampling approach used by previous studies by Brye et al. [1, 4, 7, 8] was employed in this study to characterize the short-term impacts of land leveling. The average annual rainfall of Tokat city is 443 mm, mainly distributed in winter, spring, and autumn; and the average annual temperature is 12.5°C. 

**Figure 1:**
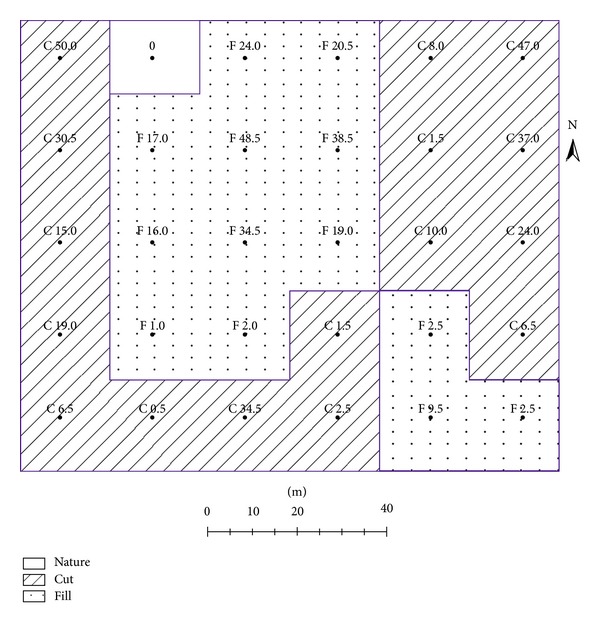
The grids of cuts or fills of the study area for land leveling (the unit of cut or fill depth in each grid is in centimeter).

 Considering both irrigation and drainage methods to be applied on the study area in the near future, the plane method [3] to determine the amounts in depths of cut or fill at each grid was employed. The slopes of the study area in both north-south and east-west directions in this method are determined by the least-squares procedure presented by Chugg [13]. Figure 1 shows the determined cut and fill grid square areas with the computed depth values of cuts and fills in centimeter. From Figure 1, the cut areas or grids were located along the South (except south-east corner), East (almost two lines along north-south direction), and west borders of the study area. While total 13 grids of 30 were determined as fill areas, 16 grids were determined as cut areas. The fill depths were ranged between 1.0 and 48.5 cm, and the cut depths were ranged between 0.5 and 50.0 cm. According to the cut and fill depths given in Figure 1 for each grid, the computed cut-fill ratio for the study area is 1.25. This computed ratio of the sum of the cuts to the sum of the fills is in the limits (1.2–1.5) recommended by James [14] and Schwab et al. [3]. After the land leveling by heavy carrier-type scraper powered with rubber tires, a 2.9% uniform slope from north to south, and 0.29% uniform slope from west to east at the study area were obtained. 

### 2.2. Soil Sampling and Measurements

 Before the leveling, the 30 grid centers (20 m apart) at the study area were setup with stakes by using a theodolite, level rods, and steel tapes in the mid of November 2010. The elevations at these centers were also measured by using these theodolite and level rods. The preleveling soil samples from both surface (0–20 cm) and subsurface soils (20–40 cm) at each of the 30 grid centers in the study area were obtained just after setting up the grids just before the land leveling activities. The land leveling activities were performed over a 2-day period (December 5-6, 2010). To characterize the changes in soil properties as a result of the land leveling, the post-leveling soil samples from both surface and subsurface soils at each of the 30 grid centers were obtained the mid of December, 2010. 

 Bulk soil samples to determine the particle size distributions, FC, PWP, WHC, pH, EC, and 100 cm^3^ (5 cm high by 5.1 cm diameter) undisturbed core samples to determine bulk densities (BDs) of surface and subsurface soils at each grid centers were used in this study. The core samples were taken vertically with a double-cylinder, hammer-driven core sampler. Bulk densities were determined as described by Blake and Hartge [15]. Bulk samples were air-dried, manually grounded, and sieved to remove coarse fragments >2 mm. The analysis of particle size distribution on the soil samples passing through 2 mm mesh screen was performed using the hydrometer method [16]. Soil water contents at the suctions of field capacity (FC) (1/3 atm.) and permanent wilting point (PWP) (15 atm.) were determined in the laboratory by ceramic plates following the procedures by Klute [17]. Soil pH and EC were determined with an electrode on a 1 : 2 soil/water solution. To determine the water holding capacity (WHC) for each soil sample, (1) was used:

(1) $$\begin{array}{c} \text{WHC}=\left(\text{FC}-\text{PWP}\right)\times \text{BD}\times D, \end{array}$$

where WHC is the water holding capacity of 2 dm depth of soil layer (mm), FC is the soil water content at the suction of field capacity (1/3 atm.) (% by weight), PWP is the soil water content at the suction of permanent wilting point (15 atm.) (% by weight), BD is the soil bulk density (g cm^−3^), and *D* is the soil depth (=2 dm).

### 2.3. Data Analysis

 Analysis of variance and subsequent statistical testing have been a common methods for comparing variations for land leveled fields. The surface subsurface soil properties (BD, sand, silt, and clay contents, pH, EC, FC, PWP, and WHC) belonging to the study area just before and after land leveling were reported as mean, standard error (SE), coefficient of variation (CV), and variance.

 Paired *t*-tests were performed to determine whether differences in the soil properties before and after land leveling are significant or not. Therefore, the results of this test shows the overall short-term effects of land leveling on the soil properties considered in this study. Employing the paired *t*-test, the means of soil properties were separated by least significant differences (LSDs) at the 0.001, 0.01, and 0.05 levels. For paired *t*-test, Minitab package program (Minitab version 12.1, Minitab, Inc., State College, PA, USA) was used. In this analysis, it was assumed that the paired differences follow a normal distribution. The effects of land leveling on the variability of the studied soil properties were also determined by Levene's test [18]. Levene's test is an inferential statistic used to assess the equality of variances in different samples. In this study, it was tested that the pre- and postleveling variances of soil properties are equal (called homogeneity of variance). Therefore, to determine the effect of land leveling on the sample variance, homogeneity of variance was evaluated using Levene's test. Variances were separated by LSD at the 0.05 level. For Levene's test, also Minitab package program was used.

 The effects of land leveling on the spatial variability of the studied soil properties were determined by geostatistical analyses. In order to determine the effects of land leveling on statistical distribution of soil properties and on the spatial relationships among soil properties before and after land leveling, geostatistical analysis can give useful information. Geostatistical analyses were conducted using GS+ (version 7.0, Gamma Design Software, Plainwell, MI). Only isotropic semivariograms were considered, and semivariance parameters of the best-fitting (i.e., smallest residual sum of squares, and the highest coefficient of determination-*r* ^2^) Gaussian, linear, spherical, or exponential models were reported for pre- and postleveled soil properties. The effects of land leveling on the spatial distributions and variability of soil properties (BD, sand, silt, clay contents, pH, EC, FC, PWP, and WHC) were analyzed by mapping pre- and post-leveling values of these properties for both surface and subsurface soils. 

 In order to explain the effects of leveling on the water holding capacities of both surface and subsurface soils of both cut and fill areas, the relationships between the changing amounts of the soil physical properties and the depths of cuts and fills through cut and fill areas were also analyzed separately by employing simple linear regression analysis.

## 3. Results and Discussion

 In this study, the land leveling resulted in an average surface elevation change of −2.5 cm (i.e., an overall cut), ranging from +18.1 cm (i.e., average fill) to −18.4 cm (i.e., average cut) across the 1.2 ha study area (Figure 1). According to the cut volume per ha area (1220 m^3 ^ha^−1^), the performed land leveling operation can be classified as a heavy leveling [19]. 

### 3.1. Changes in Soil Characteristics

 According to the paired *t*-test results, land leveling significantly affected the magnitudes of soil parameters of BD, pH, FC, PWP, and WHC for surface soil (Table 1). Land leveling also significantly affected the magnitudes of BD, clay content, pH, EC, PWP, and WHC for subsurface soil. Before the land leveling, the bulk density of surface soil ranged from 1.14 to 1.67 g cm^−3^ and averaged 1.40 (SE = 0.03 and CV = 11.01) g cm^−3^ over the 1.2 ha sampling area (Table 1). After leveling, it ranged from 1.44 to 1.85 g cm^−3^ and averaged 1.68 (SE = 0.02 and CV = 5.47) g cm^−3^. The increase in bulk density due to leveling is significant at 0.001 level. These increases in bulk density are probably due to exposure of subsoil (alluvial material) with high sand contents thereby having inherently high bulk density, soil compaction by the scraper, and arriving to a hardpan. Furthermore, land leveling decreased the overall variability of bulk density (ΔCV = −50%) in the surface soil (Table 1). Similar results with an increase of 0.08 g cm^−3^ in mean value of bulk density and a decrease of 38% in ΔCV after land leveling were also observed for subsurface soil. These kinds of increases for bulk density were also reported in the studies by Brye et al. [1, 8]. A deep tillage may be required following land leveling to help alleviate this poor soil condition. 

**Figure 2:**
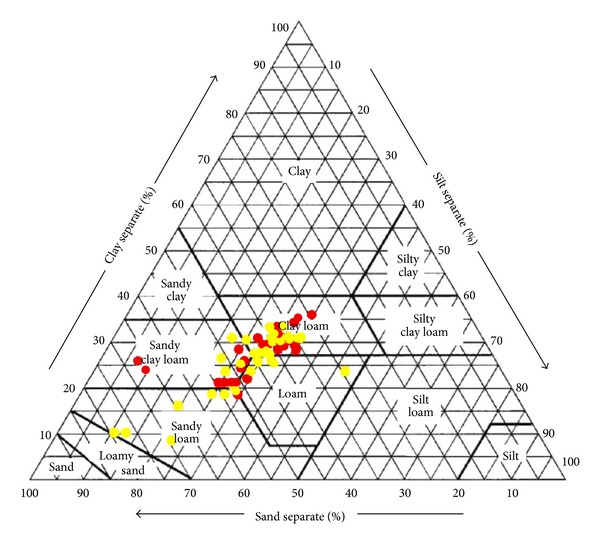
Distributions of the subsurface soil texture of study area before (red dot) and after (yellow dot) land leveling.

 When the mean values of sand, silt, and clay contents in Table 1 were used to determine soil textural classes of surface and subsurface soils before and after land leveling, the preleveled data of both surface and subsurface, and postleveled surface soils were classified as clay loam. However, the postleveled subsurface soil was classified as sandy clay loam. This change is due to probably increases of sand contents and decreases of clay contents in the deep horizons of study site soil. Furthermore, while any significant changes in soil particle size distributions of surface soil were not determined, a small decrease in clay percentage (2%) for subsurface soil was found significant (*P* ≤ 0.05). The mean sand content of subsurface soil also increased (about 3%) due to land leveling; however, this increase was not found to be significant. Textural class change of subsurface soil due to land leveling was further investigated by illustrating the sand, silt, and clay contents at each grid center on the soil texture triangle in Figure 2. From this figure, it is clear that especially four yellow-colored dots on the left bottom corner of triangle for postleveled soil are located apart from the rest of groups. The sand contents of these samples are higher, and the clay contents are lower than those of the rest of samples. Most probably the changes in textural classes of subsurface soil due to land leveling are because of these data of these samples. These samples were located in the cut areas.

 Increases in the pH values due to land leveling for both surface and subsurface soils are also significant (*P* ≤ 0.001) (Table 1). This result is different than that by Brye et al. [7]. But these increases (0.2 and 0.3 unit for surface and subsurface soils, resp.) can be considered relatively small and agronomically not significant. Furthermore, variability in the pH values was increased due to land leveling. In addition, the decrease (0.03 dS m^−1^) of EC mean for subsurface soil was also found significant (*P* ≤ 0.05) (Table 1). 

 According to the analysis of paired *t*-test, the decreases in FC and PWP, the increase in WHC values for surface soil, the decrease in PWP, and the increase in WHC values for subsurface soil were found to be significant due to land leveling. Before land leveling, FC values of surface soil ranged from 21.8 to 31.0% and averaged 26% (SE = 0.37 and CV = 7.77) (Table 1). After leveling, FC values for surface soil ranged from 19.8 to 30% and averaged 24.9% (SE = 0.53 and CV = 11.56). The decrease (1.1%) in FC due to the leveling was found significant (*P* ≤ 0.05) according to the analysis of paired *t*-test. Furthermore, the land leveling increased the overall variability of FC (ΔCV = 49%) in the surface soil. When we consider the mean values of FC after land leveling, the FC values for both surface and subsurface soils became close to each other (difference 0.5%). On the other hand, the PWP values of preleveling surface soil ranged from 14.2 to 23.5% and averaged 17.8% (SE = 0.45 and CV = 13.96). After the land leveling, the PWP values for the surface soil ranged from 10.9 to 20.3% and averaged 15% (SE = 0.35 and CV = 12.78). The decrease (2.8%) in PWP due to the leveling is significant at 0.001 level. The land leveling decreased the overall variability of PWP (ΔCV = −8.5%) in the surface soil. For subsurface soil, preleveling PWP values ranged from 10.7 to 22.7% and averaged 16.8% (SE = 0.48 and CV = 15.80). After the land leveling, the PWP values of subsurface soil ranged from 7.1 to 20.7% and averaged 15% (SE = 0.64 and CV = 23.33). The decrease (1.8%) in the PWP values of subsurface soil due to the leveling was also found significant (*P* ≤ 0.05). In contrast to the result for the surface soil, land leveling increased the overall variability of PWP (ΔCV = 48%) in the subsurface soil. Similar to the FC, after the leveling, the mean PWP values of surface and subsurface soils became equal. When we consider the WHC values which are functions of FC, PWP, and BD, the increases (10.4 and 5.2 mm for surface and subsurface soils, resp.) due to the land leveling were found to be significant at 0.001 level. The land leveling did not make a big change on the overall variability of WHC (ΔCV = 6.8% and ΔCV = 1.1% for surface and subsurface soils, resp.).

 In order to measure variability due to the land leveling, the variances of soil groups before and after land leveling were employed. As indicated, the significant changes for pre- and postleveling mean values of BD, pH, FC, PWP, and WHC for the surface soil according to the paired *t*-test (Table 1), the sample variance (*σ*) values of, same properties except PWP, were also significantly affected (*P* ≤ 0.05) by the land leveling (Table 2) according to the Levene's test. While land leveling decreased the overall variability of surface soil BD, it increased the overall variability of soil pH, FC, and WHC. Among BD, clay content, pH, EC, PWP, and WHC of the subsurface soil, only the variance values of BD and pH were significantly affected by land leveling. Similar to the results for the surface soil, the land leveling decreased the overall variability of BD of subsurface soil and it increased the overall variability of soil pH. The increases in the sample variance of pH were also reported by Brye et al. [7] and Brye [9]. As a result, according to the Levene's test, land leveling significantly affected the variance of BD, pH, FC, and WHC of the surface soil and BD and pH of the subsurface soil in this study. 

### 3.2. Changes in Spatial Variability and Distributions

 Based on the geostatistical analyses, the best-fit semivariogram models of the surface soil were changed for BD, sand content, and EC due to the land leveling (Table 3). Before the leveling, the best-fit models for surface soil parameters, except silt content, tended to have high predictive capability, where all *r* ^2^ values were ≥0.84. The best-fitting model efficiencies (*r* ^2^ values) decreased for all soil parameters (except WHC) due to leveling. After the leveling, except the FC, PWP, and WHC, the best-fit models also tended to have low predictive capability (*r* ^2^ ≤ 0.46). The Gaussian and spherical models best characterized the structures of semivariograms for surface soil FC, WHC, PWP, respectively (Table 3). Both before and after the land leveling, the model fit was very poor for the pH of the surface soil (*r* ^2^ ≤ 0.02). In addition, the model fit for the EC of postleveled surface soil was also very poor. 

 The obtained high nugget values (*C*_0_) for the sand and silt contents and WHC (Table 3) indicate microscale effects and some sampling and measurement errors. As indicated by Isaaks and Srivastava [20], the sill values (*C*_0_ + *C*) are also the variance values (Table 2) of random soil properties. Therefore, the sill values (Table 3) and variance values (Table 2) of soil properties are parallel to each other. In general, the range parameters from the best-fit semivariogram models for the surface soil properties were large (>58 m) before the leveling, indicating spatial autocorrelation among the sampling points at the 20 m spacing, and the data were not truly independent within the study area (Table 3). After the leveling, the range parameter for bulk density increased (28%). In other words, the homogeneity of bulk density across the study area increased after the land leveling. This means that after the land leveling, the bulk density is not changing more rapidly within the study area than it did before the leveling. A similar result about increase of bulk density after leveling was also stated by Brye et al. [8]. Furthermore, the range parameter for silt did not change after the leveling (Table 3). The range parameters decreased for other soil properties after the leveling. The highest decreases or changes in the range values were seen for the EC, pH, PWP, clay, and sand contents (>58%). Though the best-fit semivariogram models did not change after the leveling, the range parameters decreased about 52% and 61% for FC and PWP, respectively. The clay content, pH, EC, FC, and PWP achieved spatial independence (i.e., the range parameter < 36 m) within the study area due to land leveling (Table 3). These changes may be the causes of immediate disruption of a previous quasi-equilibrium, in which land leveling activities imparted on the soil can have lasting negative effects on soil properties [1, 4, 7, 8]. 

 For surface soil, while the PWP had a significant spatial component (*C*) before land leveling, PWP also had a significant spatial component with a little bit decreasing significance order after land leveling (Table 3). Another significant spatial component was observed for the FC of surface soil after the land leveling. The proportion *C*/(*C*_0_  +  *C*), representing inherent variability in the data, explained the highest variability (99.9%) in PWP and no variability in pH (0%) of the surface soil before the leveling. The proportion values of other preleveling surface soil properties, except the silt content, are high (>60%). These results are parallel to the values of *r* ^2^ (predictive capability of the best-fit model). After leveling, the 20 m spacing appeared too large to ascertain a spatial dependency for the BD and EC within the sampling area. Furthermore, after the leveling, the inherent variability values explained also large fractions of the total variability in the postleveled soil properties as it did for the soil properties of clay content, pH, FC, PWP, and WHC before the leveling.

 The best-fit semivariogram models of subsurface soil were changed for all soil properties, except silt content, due to land leveling (Table 4). After leveling, the best-fit semivariogram models were linear for all the soil properties, except PWP (spherical). The best-fit models for the subsurface soil properties tended to have high predictive capability (*r* ^2^ ≥ 0.83) before leveling. The best-fitting model efficiencies (*r* ^2^ values) decreased for the soil parameters (except silt content) due to leveling. The model fit for the BD of postleveled subsurface soil was very poor (*r* ^2^ = 0.01). After leveling, except the clay content, and pH, the best-fit models also tended to have high predictive capability (*r* ^2^ ≥ 0.57). The model fits were poor for the clay content and pH of the postleveled subsurface soil (*r* ^2^ ≤ 0.45). While the Gaussian model best characterized the structures of semivariograms for the subsurface soil FC, WHC, and PWP before leveling, the linear and spherical models best characterized the structures of semivariograms for the postleveled subsurface soil FC, WHC, and PWP, respectively. 

 Similar to those of the surface soil, the high nugget values (*C*_0_) for the sand and silt contents and WHC of subsurface soil before leveling; for the sand, silt and clay contents FC, and WHC of the postleveled subsurface soil were also obtained (Table 4). Both pre- and post-leveling range values from the best-fit semivariogram models for the subsurface soil properties are large (≥55 m). Unlike to the surface soil, the land leveling did not cause much spatial independence to the subsurface soil properties. After leveling, small increases in the range values of BD and sand and silt contents and small decreases in the range values of FC, PWP, and WHC were obtained. The decrease in the range value of EC is high (51%). The range parameters for the silt and pH did not change after leveling (Table 4). 

 The properties of subsurface soil did not have any significant spatial component (*C*). Before leveling and the proportion values, *C*/(*C*_0_ + *C*), explained between 56 and 87% of the variation in soil properties, except silt content (35%) (Table 4). In contrast, the 20 m spacing appeared too large to ascertain a spatial dependency for the BD, pH, and EC of the postleveled subsurface soil within the sampling area. Due to leveling, the amount of variation in the FC and WHC, explained by the proportion column, decreased to 43 and 30%, respectively. The amount of variation explained by the proportional spatial component following leveling also decreased by 28% for the PWP.

 The spatial distributions of all soil properties measured in the both surface (0–20 cm) and subsurface (20–40 cm) soils were altered by land leveling. While the areas that were initially cut and the areas that were initially fill were given in Figure 1, they also can be observable from the figures of spatial distributions of soil properties with the help of Figure 1. However, it will be difficult to associate exactly the changes according to the initial cut and fill areas. 

**Figure 3:**
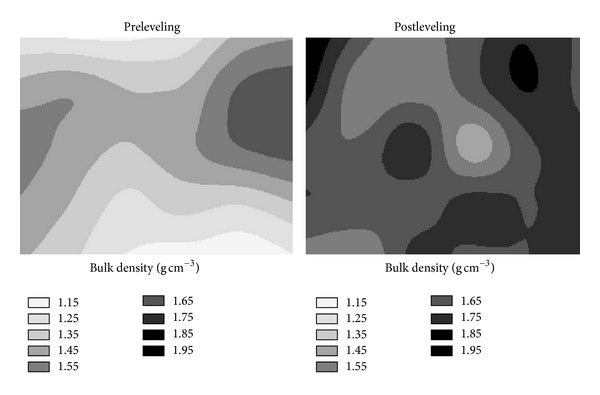
Pre- and post-leveling spatial distributions of soil bulk density for surface soil (0–20 cm).

 Almost the all locations within the study area with the low bulk densities before leveling had noticeably high bulk densities after leveling (Figure 3). In general, the cut areas before and after leveling had higher bulk densities than that of fill areas due to most probably exposure of the subsoil that had higher bulk density than that of the original surface soil, soil compaction by machinery, and arriving to a hardpan. Similar results are valid for the bulk densities of subsurface soil (spatial distribution map of bulk density for subsurface soil was not given in order to save space in the paper). 

**Figure 4:**
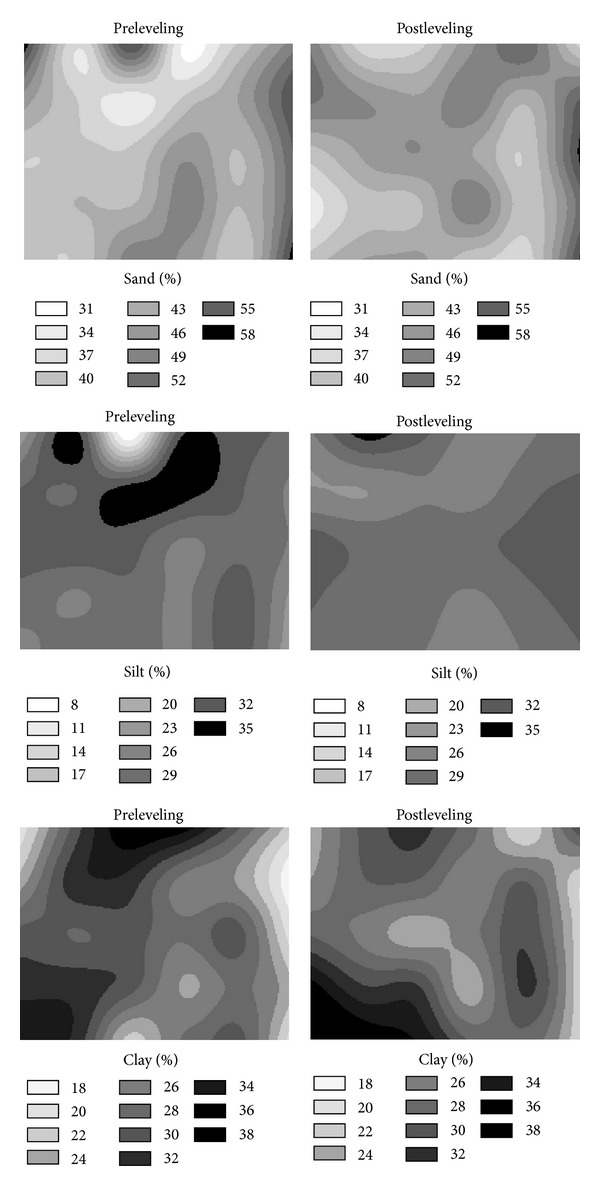
Pre- and post-leveling spatial distributions of soil particle-size fractions in surface soil (0−20 cm).

 Changes in the spatial distributions of sand, silt, and clay contents of the surface soil also occurred (Figure 4). Some locations with high sand, silt, and clay contents became with slightly low sand, silt, and clay contents, or vice versa. Especially the sand content increases are high in the fill areas. While the silt contents of the couple of locations of study area decreased after leveling, in general, small increases in silt contents at the majority of other locations were seen. After leveling, the spatial distribution of silt content of the study area became more homogeneous as it was also indicated by the CV values in Table 1. Furthermore, a small increase in the homogeneity of clay content spatial distribution over the study area after leveling was also noticed. In general, the clay contents of the fill areas decreased (Figure 4). As a result, small, not significant alterations on the soil particle size fractions in the surface soil were noticed in this study. This is most probably due to exposure of and mixing with subsoil with similar particle size fractions of surface soil. This result is different to those of Brye et al. [1, 8]. Increases in the sand contents especially on three grid centers of the cut areas and not much change of the sand contents in the fill areas were observed for the subsurface soil after leveling (not shown). After leveling, small decreases in the clay contents of the subsurface soil at the cut areas were also noticed. 

**Figure 5:**
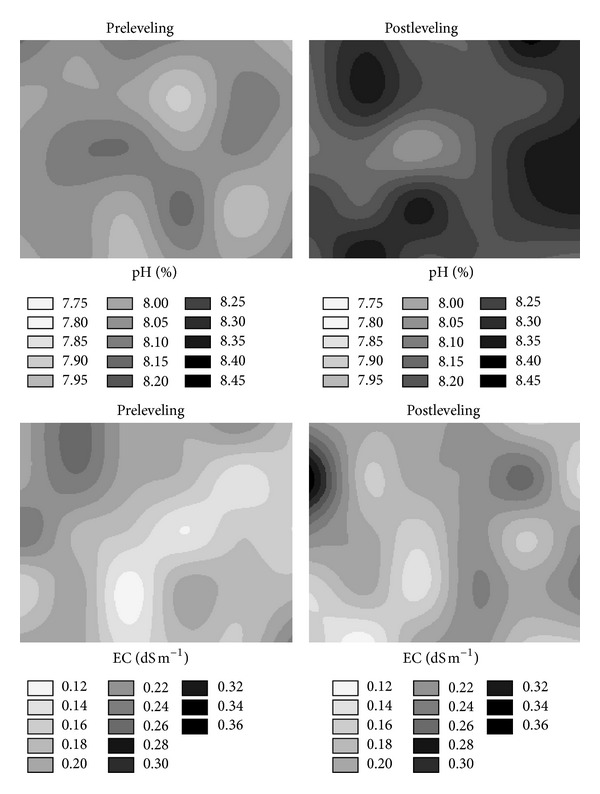
Pre- and post-leveling spatial distributions of soil pH, electrical conductivity (EC), and available water holding capacity (AWHC) in surface soil (0–20 cm).

 The spatial distribution maps of pH and EC of the surface soil were altered to some degree by the land leveling (Figure 5). The pH values of the postleveled surface soil for almost all locations increased. In addition, the spatial distribution of pH values at the middle part of the fill areas seems to have the lowest pH values after the leveling. Similar increases of the pH values after the leveling for the subsurface soil were also observed (not shown). As a result of the land leveling, the soil of the study area became slightly more alkaline. The increases or decreases of pH for surface soil after leveling were also stated by Brye et al. [7, 8]. [The authors reported that exposing and mixing alkaline surface soil with the typically acidic or alkaline subsoil as reasons for these changes on the pH.] On the other hand, small increases or decreases in the EC values on some locations of the study area caused differences in the spatial distribution of the EC values for the surface soil. In general, the low EC values were located in the fill areas. On the other hand, in general, the EC values of the subsurface soil over the study area, except three locations or grids, decreased after the leveling (not shown). These results of EC changes after the leveling are different from those by Brye et al. [7, 8]. 

**Figure 6:**
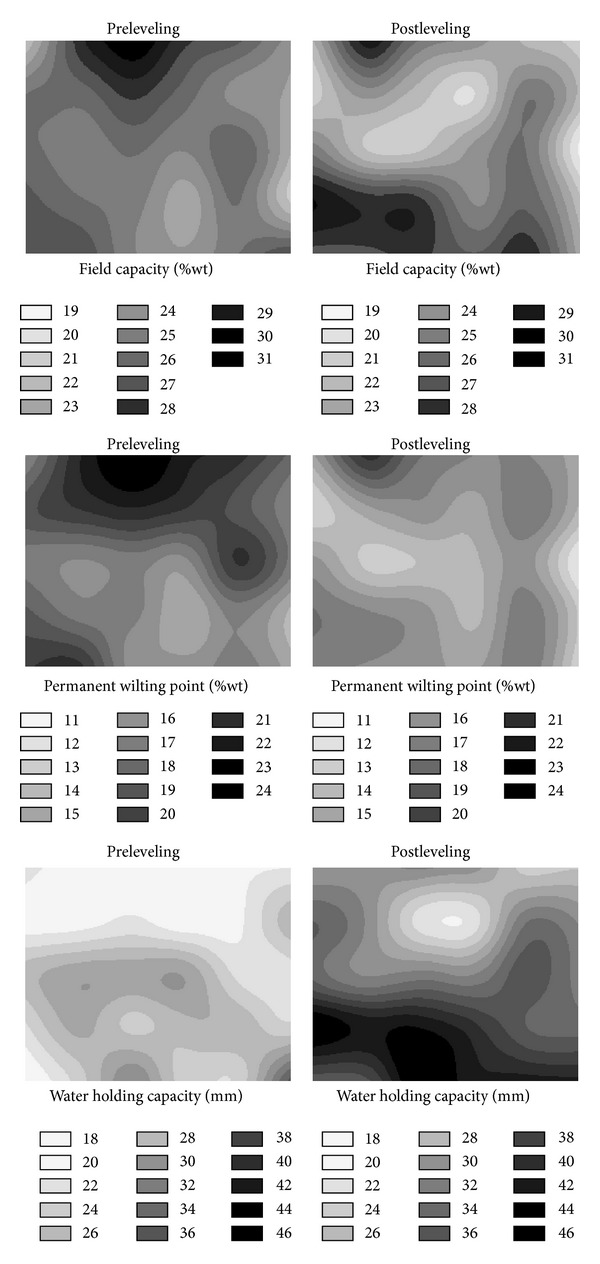
Pre- and post-leveling spatial distributions of soil field capacity, permanent wilting point, and water holding capacity for surface soil (0–20 cm).

**Figure 7:**
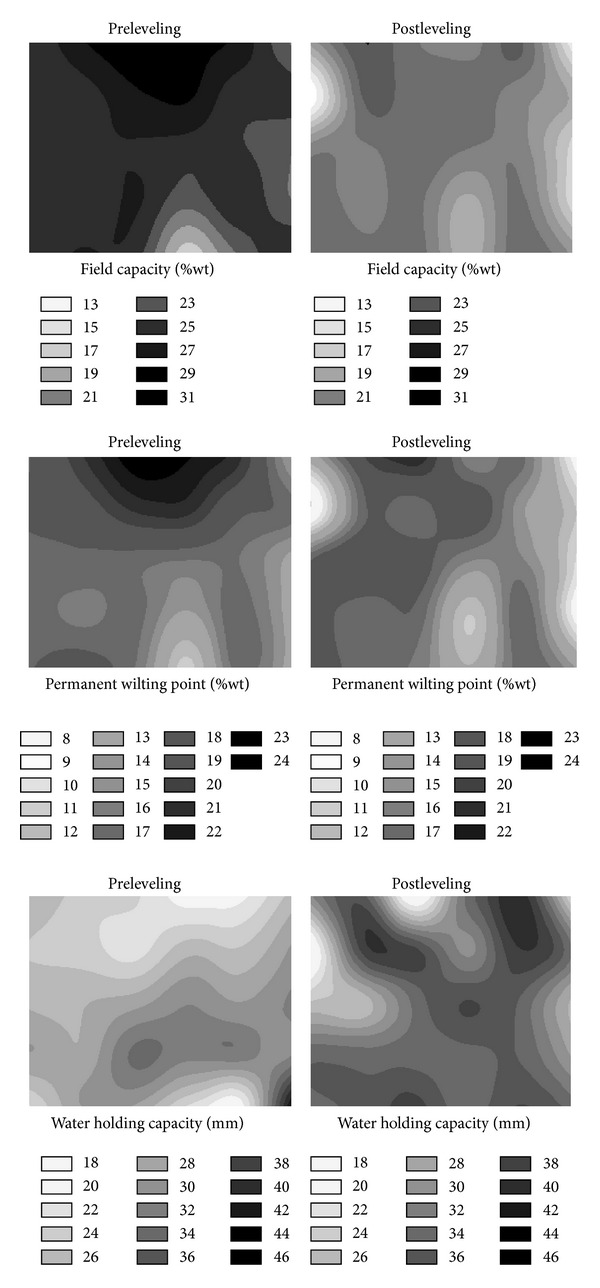
Pre- and post-leveling spatial distributions of soil field capacity, permanent wilting point, and water holding capacity for subsurface soil (20–40 cm).

 The spatial distributions of the FC, PWP, and WHC of both the surface and subsurface soils also changed as a result of land leveling (Figures 6 and 7). The distribution of FC values over the study area before leveling was much more homogeneous than that after leveling (Figure 6). After the leveling, the FC values for almost all locations, except a couple of locations on the south-west portion of the study area, slightly decreased. It seems that the decreases in the fill areas were higher than those from the cut areas. After leveling, the PWP values of the surface soil over almost all locations of the study area decreased (Figure 6). The reasons for these decreases of both FC and PWP values can be the slight decreases of clay contents and slight increases of sand contents of surface soil after leveling. In contrast to the FC and PWP values, the WHC values increased over almost all grids of the research area after leveling (Figure 6). It looks like the fill areas located in the middle part of the research area had the lowest WHC values after leveling. As it can be inferred from (1), WHC is a function of FC, PWP, and BD. Therefore, the increases of WHC are due to high differences between the FC and PWP values with the high BD values. While we expected a more uniform spatial distribution of WHC values over the research area after leveling, it did not happen. Therefore, this kind of area may need much more attention especially for irrigation planning and management. Maybe, site specific irrigation planning and management can be an alternative for sustainable agriculture or research on these kinds of areas.

 After the land leveling, decreases of the FC values of subsurface soil (20–40 cm) over almost all locations of the study area can be seen in Figure 7. Furthermore, it seems that the spatial distribution of the FC values before leveling was more homogeneous than that after leveling. In addition, both before and after leveling, the FC values in the fill areas are higher than those of the cut areas. The decreases of FC values in the cut locations may be due to exposure of subsoil with the high sand contents. The PWP values of the subsurface soil showed similar spatial distribution to that of the FC of subsurface soil for both before and after levelings (Figure 7). In general, the locations had the high FC values also had the high PWP values; and the locations that had the low FC values also had the low PWP values. Similar to the FC values, the PWP values of subsurface soil (20–40 cm) over almost all locations of the study area decreased after leveling (Figure 7). The WHC values of subsurface soil also increased as the WHC values of surface soil as a result of the land leveling (Figure 7).

### 3.3. Relationships between the Amounts of Changes in Soil Physical Properties and Depths of Cut and Fill

**Figure 8:**
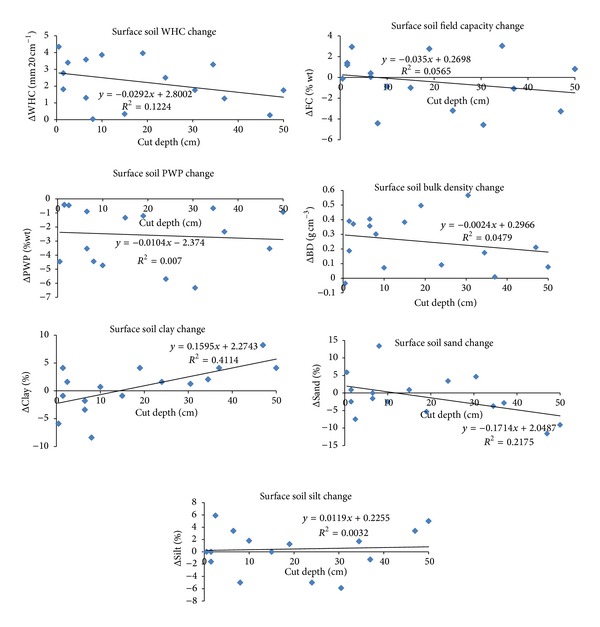
The relationships between the amounts of change of surface soil (0–20 cm) physical properties that occurred as a result of leveling and cut depths through cut areas.

**Figure 9:**
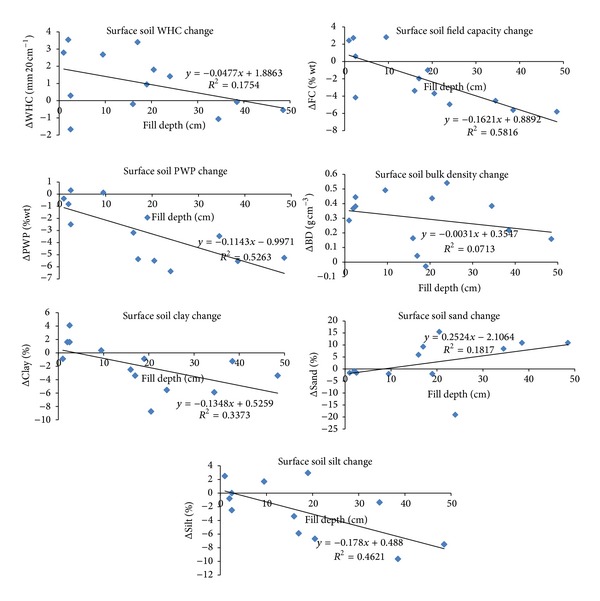
The relationships between the amounts of change of surface soil (0–20 cm) physical properties that occurred as a result of leveling and fill depths through fill areas.

The mean values of changes for the soil properties of cut and fill areas were given in Table 5 separately. The result of the overall decreases in both FC and PWP values and the increases in BD values due to leveling, which caused the increases in WHC values, can also be induced from the given values in Table 5. As stated earlier, the reasons for the increases in WHC values due to leveling in both cut and fill areas of both surface and subsurface soils [are look like the much more decreases] in PWP values than those of FC values and the increases in BD values. The overall WHC increase in the cut areas (29%) occurred more than twice of that in the fill areas (12%) for the surface soil of the study area. However, the overall WHC increase in the cut areas (13%) is close to that in the fill areas (15%) for the subsurface soil of the study area. In addition, the reasons that caused the decreases in both FC and PWP and the increases in BD may be mainly due to the decreases of clay and increases of sand contents of both cut and fill subsurface and fill surface soils (Table 5). The values of increases or decreases (change amounts) against the cut depths through cut areas and the fill depths through fill areas are presented in Figures 8 and 9, respectively. The situation of overall decreases of clay and increases of sand contents [looks like did not resulted] for the surface soils through cut areas mainly because of the high increases of clay and high decreases of sand contents when the cut depth is greater than 30 cm (Figure 8). The WHC values in all grids of cut area (Figure 8) and in 2/3rd of grids of fill area (Figure 9) of surface soil increased (above horizontal axes) due to leveling. In addition, increases of the WHC values in 2/3rd of grids of both cut and fill areas (not given) of subsurface soil were also observed. The decreases of PWP and increases of BD values of surface soil in almost all grids of both cut and fill areas are also clear from these figures. 

 The results of linear relationships with the values of their importance (*R* ^2^) between the amounts of changes in the soil physical properties due to the leveling and the depths of cut and fill are also presented in Figures 8 and 9 for surface soils through the cut and fill areas separately. Some important results from this analysis are as follows: the surface soil clay contents through cut areas increased as cut depths increased (*r* = 0.64) (Figure 8), the surface soil FC and PWP values through fill areas decreased as fill depths increased (*r* = 0.76, and *r* = 0.73, resp.) (Figure 9), and the surface soil clay and silt contents through fill areas decreased as fill depths increased (*r* = 0.58 and *r* = 0.68, resp.) (Figure 9). We did not determine any moderate or strong relationships between the changing amounts of the subsurface soil physical properties due to leveling and the depths of cut and fill. 

## 4. Conclusions

 When comparing some irrigation planning and management related soil properties (bulk density, particle size distributions, pH, electrical conductivity, field capacity, permanent wilting point, and water holding capacity) of a clay loam soil before and just after land leveling, we saw that the practice of land leveling significantly affected the magnitude, spatial variability, and spatial distribution of these soil properties in both surface (0–20 cm) and subsurface (20–40 cm) clay loam soils. Increased bulk density, pH, and water holding capacity and decreased permanent wilting point due to land leveling are some of the results of this study. The moderate negative relationships between the surface soil FC and PWP values and fill depths and between the clay and silt contents and fill depths through fill areas were found. Further research about these kinds of relationships may be needed in larger study areas. 

 The study showed that while land leveling is an agricultural practice to facilitate more uniform distribution of irrigation water, if leveling is poor, then changes in soil properties are notable and may be detrimental when observed at a short time after the operation. Applying equal amount of irrigation water on overall land with heterogenic soil properties alone will not be enough for productivity. After leveling, some other site specific cautions for defining and refining management practices to regain productivity and for improving homogeneity in soil properties are needed. In addition, research about long-term effects of land leveling with an adapted land reclamation policy to improve homogeneity in soil properties may be needed. 

## Figures and Tables

**Table 1:** The effect of land leveling on some selected soil properties from both 0–20 cm (surface soil) and 20–40 cm (subsurface soil). Means, standard errors, coefficients of variation, and the changes in coefficients of variation (ΔCV) of pre- and postleveling soil properties for both surface and subsurface soils were reported. Asterisks next to postleveling means represent significant differences between pre- and postleveling measurements caused by land leveling. These asterisks were determined after paired *t*-test (*n* = 30).

Soil parameter	Surface soil							Subsurface soil						
	Preleveling			Postleveling			ΔCV^+^ (%)	Preleveling			Postleveling			ΔCV (%)
	Mean	S. error	CV_pre_	Mean	S. error	CV_post_		Mean	S. error	CV_pre_	Mean	S. error	CV_post_	
BD (g cm^−3^)	1.40	0.03	11.01	1.68***	0.02	5.47	−50	1.59	0.02	7.67	1.67***	0.01	4.73	−38
Sand (%)	43.88	1.37	17.16	44.36	1.17	14.50	−16	44.00	1.42	17.73	46.88	2.24	26.22	48
Silt (%)	28.33	0.98	18.87	28.33	0.59	11.39	−40	28.02	0.87	17.07	27.44	1.19	23.83	40
Clay (%)	27.79	0.94	18.46	27.31	0.72	14.36	−22	27.72	0.85	16.74	25.67*	1.29	27.54	65
pH	8.02	0.01	0.74	8.21***	0.02	1.08	45	7.92	0.02	1.12	8.21***	0.03	1.82	63
EC (dS m^−1^)	0.187	0.01	18.66	0.198	0.01	21.90	17	0.246	0.01	23.49	0.216*	0.01	27.37	17
FC (%wt)	26.0	0.37	7.77	24.9**	0.53	11.56	49	25.1	0.46	9.99	24.4	0.80	17.94	80
PWP (%wt)	17.8	0.45	13.96	15.0***	0.35	12.78	−8.5	16.8	0.48	15.80	15.0**	0.64	23.33	48
WHC (mm)	22.7	0.76	18.40	33.1***	1.19	19.66	6.8	26.2	0.87	18.25	31.4***	1.06	18.45	1.1

+: ΔCV = 100 × [CV_post_ − CV_pre_]/CV_pre_.

∗: 0.01 < *P* ≤ 0.05.

∗∗: 0.001 < *P* ≤ 0.01.

∗∗∗: *P* ≤ 0.001.

**Table 2:** The effects of land leveling on the sample variances of some selected soil properties from both 0–20 cm (surface soil) and 20–40 cm (subsurface soil). Asterisks next to postleveling variance values represent significant differences in the sample variances of pre- and postleveling soil properties caused by land leveling. The asterisks were obtained after employing Levene's test (*n* = 30).

Soil parameter	Surface soil		Subsurface soil	
	Preleveling	Postleveling	Preleveling	Postleveling
BD (g cm^−3^)	0.024	0.008*	0.015	0.006*
Sand (%)	56.7	41.3	60.8	151.0
Silt (%)	28.6	10.4	22.9	42.8
Clay (%)	26.3	15.4	21.5	50.0
pH	0.004	0.008*	0.008	0.022*
EC (dS m^−1^)	0.001	0.002	0.003	0.003
FC (%wt)	4.07	8.30*	6.26	19.13
PWP (%wt)	6.17	13.69	7.06	12.20
WHC (mm)	17.42	42.25*	22.89	35.52

∗: *P* ≤ 0.05.

**Table 3:** Summary of geostatistical parameters for surface soil (0–20 cm) properties measured before and after land leveling.

Soil parameter	Preleveling						Postleveling					
	Model	Nugget (*C*_0_)^†^	Sill (*C*_0_ + *C*)^†^	Range (m)	*C*/(*C*_0_ + *C*)	*r* ^2^	Model	Nugget (*C*_0_)	Sill (*C*_0_ + *C*)	Range (m)	*C*/(*C*_0_ + *C*)	*r* ^2^
BD (g cm^−3^)	Gaussian	0.0092	0.0262	58.5	0.651	0.98	Linear	0.0085	0.0085	74.6	0.000	0.46
Sand (%)	Gaussian	38.90	98.95	183.4	0.607	0.94	Linear	31.03	40.12	74.6	0.227	0.46
Silt (%)	Linear	25.45	32.03	74.6	0.206	0.42	Linear	8.36	10.72	74.6	0.220	0.30
Clay (%)	Spherical	3.44	30.23	83.0	0.886	0.99	Spherical	0.20	14.13	28.0	0.986	0.39
pH	Linear	0.004	0.004	74.6	0.000	0.00	Spherical	0.00	0.0077	23.3	0.999	0.02
EC (dS m^−1^)	Spherical	0.0005	0.0014	69.4	0.609	0.84	Linear	0.00	0.0016	20.0	0.000	0.06
FC (%wt)	Gaussian	1.66	5.015	73.3	0.668	0.99	Gaussian	0.01	8.35	35.2	0.999	0.87
PWP (%wt)	Spherical	0.01	8.09	80.2	0.999	0.99	Spherical	0.01	3.54	31.5	0.997	0.56
WHC (mm)	Gaussian	8.86	36.15	158.7	0.755	0.93	Gaussian	10.7	68.78	98.0	0.844	0.99

†: *C*_0_ represents the inherent nonspatially related variability in the data, and *C* represents the variability explained by any spatial component in the data.

**Table 4:** Summary of geostatistical parameters for subsurface soil (20–40 cm) properties measured before and after land leveling.

Soil parameter	Preleveling						Postleveling					
	Model	Nugget (*C*_0_)^†^	Sill (*C*_0_ + *C*)^†^	Range (m)	*C*/( *C*_0_ + *C*)	*r* ^2^	Model	Nugget (*C*_0_)	Sill (*C*_0_ + *C*)	Range (m)	*C*/(*C*_0_ + *C*)	*r* ^2^
BD (g cm^−3^)	Gaussian	0.0054	0.019	69.9	0.718	0.97	Linear	0.006	0.0063	74.6	0.051	0.01
Sand (%)	Spherical	23.50	65.82	68.8	0.643	0.91	Linear	92.59	152.9	74.6	0.394	0.67
Silt (%)	Linear	16.87	25.81	74.6	0.347	0.83	Linear	27.4	48.02	74.6	0.435	0.88
Clay (%)	Spherical	5.02	21.71	55.0	0.769	0.84	Linear	33.31	46.27	74.6	0.280	0.43
pH	Spherical	0.003	0.009	75.6	0.670	0.96	Linear	0.0232	0.0232	74.6	0.000	0.45
EC (dS m^−1^)	Gaussian	0.0018	0.0066	151.6	0.723	0.99	Linear	0.0038	0.0038	74.6	0.000	0.93
FC (%wt)	Gaussian	2.44	9.339	90.6	0.739	0.99	Linear	11.52	20.16	74.6	0.429	0.80
PWP (%wt)	Gaussian	1.50	11.21	92.5	0.866	0.99	Spherical	4.79	12.60	73.1	0.620	0.81
WHC (mm)	Gaussian	11.66	26.82	93.4	0.565	0.95	Linear	25.30	36.28	74.6	0.302	0.57

†: *C*_0_ represents the inherent nonspatially related variability in the data, and *C* represents the variability explained by any spatial component in the data.

**Table 5:** Mean changes in surface and subsurface soil properties of cut and fill areas separately that occurred as a result of land leveling.

Area	Soil depth (cm)	WHC (mm)	FC (%wt)	PWP (%wt)	BD (g cm^−3^)	Clay (%)	Sand (%)	Silt (%)	EC (dS m^−1^)	pH
Cut	0–20	2.26	−0.37	−2.57	0.25	0.66	−1.10	0.44	22.81	0.21
	20–40	1.19	−1.51	−2.70	0.04	−2.77	5.00	−2.25	−16.81	0.28
Fill	0–20	1.02	−2.05	−3.07	0.30	−1.92	2.47	−0.55	2.47	0.16
	20–40	1.06	0.17	−0.89	0.12	−1.52	0.64	0.82	−34.73	0.30

## References

[1] Brye KR, Slaton NA, Savin MC, Norman RJ, Miller DM (2003). Short-term effects of land leveling on soil physical properties and microbial biomass. *Soil Science Society of America Journal*.

[2] Unger PW, Fulton LJ, Jones OR (1990). Land-leveling effects on soil texture, organic matter content, and aggregate stability. *Journal of Soil & Water Conservation*.

[3] Schwab GO, Fangmeier DD, Elliot WJ, Frevert RK (1993). *Soil and Water Conservation Engineering*.

[4] Brye KR, Slaton NA, Norman RJ (2005). Penetration resistance as affected by shallow-cut land leveling and cropping. *Soil and Tillage Research*.

[5] Eck HV (1987). Characteristics of exposed subsoil-at exposure and 23 years later. *Agronomy Journal*.

[6] Walker TW, Kingery WL, Street JE (2003). Rice yield and soil chemical properties as affected by precision land leveling in alluvial soils. *Agronomy Journal*.

[7] Brye KR, Slaton NA, Mozaffari M, Savin MC, Norman RJ, Miller DM (2004). Short-term effects of land leveling on soil chemical properties and their relationships with microbial biomass. *Soil Science Society of America Journal*.

[8] Brye KR, Slaton NA, Norman RJ (2006). Soil physical and biological properties as affected by land leveling in a clayey aquert. *Soil Science Society of America Journal*.

[9] Brye KR (2006). Soil biochemical properties as affected by land leveling in a clayey aquert. *Soil Science Society of America Journal*.

[10] Ramos MC, Cots-Folch R, Martínez-Casasnovas JA (2007). Effects of land terracing on soil properties in the Priorat region in Northeastern Spain: a multivariate analysis. *Geoderma*.

[11] Taşova H (1992). *Tokat Ziraat Fakültesi Yerleşim Alanının Toprak Etüt, Haritalanması ve Sınıflandırılması [M.S. thesis]*.

[12] Soil Survey Staff (1999). *Keys to Soil Taxonomyedition*.

[13] Chugg GE (1947). Calculations for land gradation. *Agricultural Engineering*.

[14] James LG (1993). *, Principles of Farm Irrigation System Design*.

[15] Blake GR, Hartge KH, Klute A (1994). Bulk density. *Methods of Soil Analysis, Part 1, Agronomy*.

[16] Gee GW, Bauder JW, Klute A (1994). Particle-size analysis. *Methods of Soil Analysis, Part 1, Agronomy*.

[17] Klute A, Klute A (1994). Water retention: laboratory methods. *Methods of Soil Analysis, Part 1. Agronomy*.

[18] Levene H (1960). *Contributions to Probability and Statistics*.

[19] Güngör Y, Yıldırım O (1989). *Tarla Sulama Sistemleri*.

[20] Isaaks EH, Srivastava RM (1989). *Applied Geostatistics*.

